# Need for the Development of a Specific Regulatory Framework for Evaluation of Mobile Health Apps in Peru: Systematic Search on App Stores and Content Analysis

**DOI:** 10.2196/16753

**Published:** 2020-07-10

**Authors:** Leonardo Rojas Mezarina, Javier Silva-Valencia, Stefan Escobar-Agreda, Daniel Hector Espinoza Herrera, Miguel S Egoavil, Mirko Maceda Kuljich, Fiorella Inga-Berrospi, Sergio Ronceros

**Affiliations:** 1 Unidad de Telesalud, School of Medicine Universidad Nacional Mayor de San Marcos Lima Peru; 2 School of Medicine Universidad San Martin de Porres Lima Peru; 3 Universidad Continental Lima Peru; 4 Deanery of the School of Medicine Universidad Nacional Mayor de San Marcos Lima Peru

**Keywords:** mhealth apps, mHealth, regulatory framework, Peru, eHealth

## Abstract

**Background:**

In Peru, there is an increase in the creation of mobile health (mHealth) apps; however, this situation could present problems related to the quality of information these apps share, data security and privacy, usability, and effectiveness, as there is no specific local regulation about their creation and use.

**Objective:**

The objective of this study was to review mHealth apps created, uploaded, or used in Peru, and perform an analysis of the national regulatory framework that could be applied to evaluate whether there is a need to develop and implement a specific regulation to these apps.

**Methods:**

A total of 3 reviews were performed. First, we reviewed information about Peruvian mHealth apps created up to May 2019 from scientific publications, news, government communications, and virtual stores, and evaluated their purpose, creator, and the available evidence of their usability and effectiveness. The second review was carried out by taking a sample of the 10 most commonly used mHealth apps in Peru (regardless of the country of creation), to evaluate the information they collect and classify them according to the possible risks that they could present in terms of security and privacy. In addition, we evaluated whether they refer to or endorse the information they provided. Finally, in the third review, we searched for Peruvian standards related to electronic health (eHealth) that involve information technology that can be applied to regulate these apps.

**Results:**

A total of 66 apps meeting our inclusion criteria were identified; of these, 47% (n=31) belonged to government agencies and 47% (n=31) were designed for administrative purposes (private and government agencies). There was no evidence about the usability or effectiveness of any of these apps. Concerning the 10 most commonly used mHealth apps in Peru, about the half of them gathered user information that could be leaked, changed, or lost, thus posing a great harm to their users or to their related patients. In addition, 6/10 (60%) of these apps did not mention the source of the information they provided. Among the Peruvian norms, the Law on the Protection of Personal Data, Law on Medical Devices, and administrative directives on standards and criteria for health information systems have some regulations that could be applied to these apps; however, these do not fully cover all aspects concerning the evaluation of security and privacy of data, quality of provided information, and evidence of an app’s usability and effectiveness.

**Conclusions:**

Because many Peruvian mHealth apps have issues related to security and privacy of data, quality of information provided, and lack of available evidence of their usability and effectiveness, there is an urgent need to develop a regulatory framework based on existing medical device and health information system norms in order to promote the evaluation and regulation of all the aforesaid aspects, including the creation of a national repository for these apps that describes all these characteristics.

## Introduction

Mobile apps are any software programs that can be downloaded and executed on a mobile device such as cell phones or tablets [1]. Since their large-scale introduction approximately 10 years ago, apps have been greatly developed and diversified, taking on a leading role in practically all the daily activities that we carry out [2]. When apps are related to aspects of health, these are called *mobile medical apps* [3], *mobile apps in health*, or simply *apps in health*. Such apps are within the technological branch known as mobile health (mHealth), which is defined as the practice of public health using mobile devices to improve health and medical care through new forms of interactive services that promote wellness, care, prevention, and disease management [4,5].

mHealth apps are classified into 2 large groups: apps for health personnel and apps for general public. Those directed at health personnel can be used for training, consultations, communication between professionals, referrals, monitoring, or patient management [6], whereas those aimed at the general public are related to medication management; symptoms of chronic diseases; or monitoring of aspects such as weight, obesity, nutrition, fertility, lactation, and pregnancy [6]. Therefore, the use of mHealth apps could aid in achieving better disease control, changing habits, improving the quality of care, allowing greater access to information [2], reducing costs, avoiding unnecessary medical consultations, and bringing health services closer to people.

Nevertheless, the possible risks of the indiscriminate creation and use of mHealth apps are worrisome [7]. Many studies have reported limited or inconclusive results regarding their effectiveness, which is mainly because it is impossible to formulate a successful and adequate design strategy for medical apps [8]; or in some cases, the effect achieved cannot be attributed solely to the use of these apps [9]. In addition, doubts have been raised about the quality of the information they provide that would be used for promotion of health, prevention of diseases, or in some cases, even management and treatment of diseases. Finally, there has been an increasing interest in evaluating the data privacy and security policies of those apps, which when left unmonitored pose a huge risk to their users [10]. To manage these situations, some international institutions such as the US Food and Drug Administration (FDA) and the European Union (EU) have developed standards and norms to regulate the development and use of mHealth apps [3].

In Peru, given the steady increase in the rate of access to mobile phones in the last few years, a need arose to promote the development of mHealth apps, as in other developing countries [11], through different strategies such as hackathons (ie, meetings for collaborative development) [12] and funding calls to support information and communications technology projects by public and private institutions [13]. However, it remains unknown whether the current Peruvian norms adequately regulate aspects related to the creation and use of mHealth apps or whether there is a need to implement a specific regulatory framework for this purpose. Therefore, in this study, we review all mHealth apps created and published in Peru and perform an analysis of the current Peruvian norms that could be applied for their regulation, in order to evaluate whether there is a need to implement a specific mHealth regulatory framework.

## Methods

### Overview

A total of 3 reviews were performed up to May 2019. The first was a review of mHealth apps created in Peru, the second was a review on mHealth apps most used, and the third was a study on the Peruvian regulatory aspects that could be applied to mHealth.

### Review of Mobile Apps Created in Peru

#### Data Sources

A search for information about Peruvian mHealth apps was performed on scientific publications, gray literature, and app stores. For the scientific literature review, the following databases were searched: PubMed, SciELO, Scopus, IEEE Xplore, and Web of Knowledge (Web of Science); for the gray literature search, we reviewed newspapers, reports of research institutes and nongovernmental organizations, thesis, conference proceedings, and websites (identified through Google Search); and concerning medical apps published in virtual app stores, we searched only Google Play and Apple Store, as most smartphones used in Peru run on either Android or iOS (Table 1).

**Table 1:** Search strategy.

Search strategy/Review employed	Data sources
Scientific literature review	Bibliographic database and search engines: PubMed, SciELO, Scopus, IEEE Xplore, Web of Knowledge (Web of Science), Google Scholar, and Google Search.
Gray literature	Peruvian National Digital Repository of Science, Technology and Innovation, Library of Theses and Dissertations (ALICIA) Newspapers: Correo, El Comercio, El Peruano, Expreso, Primera, Razón, La Republica, Gestión, Ojo, and Perú21 Websites: gob.pe/minsa, dge.gob.pe, web.ins.gob.pe, nongovernmental organizations Proceedings from related conferences Not-for-profit organizations websites Blogs and other networks
Virtual mobile app stores	Google Play: medical apps Apple App Store: medical apps

#### Search Methods

To search mHealth apps on app stores, the following search terms in Spanish were included: health, Perú, Peruvian, disease, patient care, drugs, pharmacy, insurance, self-care, Clinics, Hospitals, MINSA, DIGESA, INS, SuSalud, EsSalud, DIRESA, and SIS.

For the search in scientific and gray literature, we used 30 keywords in Spanish and English to identify apps published up to May 9, 2019. These keywords included the following:

- Peru-related keywords: related to Peru or Peruvian institutions. For example, Peru, Ministry of Health (or its acronym in Spanish MINSA), National Institute of Health (or its acronym in Spanish INS).
- Health-related keywords: Related to apps associated with providing health services for education, prevention, diagnosis, or treatment purposes. For example, patient care, self-care, health services, diagnosis.
- Mobile technology–related keywords: Related to the use of mHealth technology. For example, mobile, mHealth, app, mobile software.

Additional details about the search algorithms used are presented in Multimedia Appendix 1.

#### Eligibility Criteria

**Figure 1:**
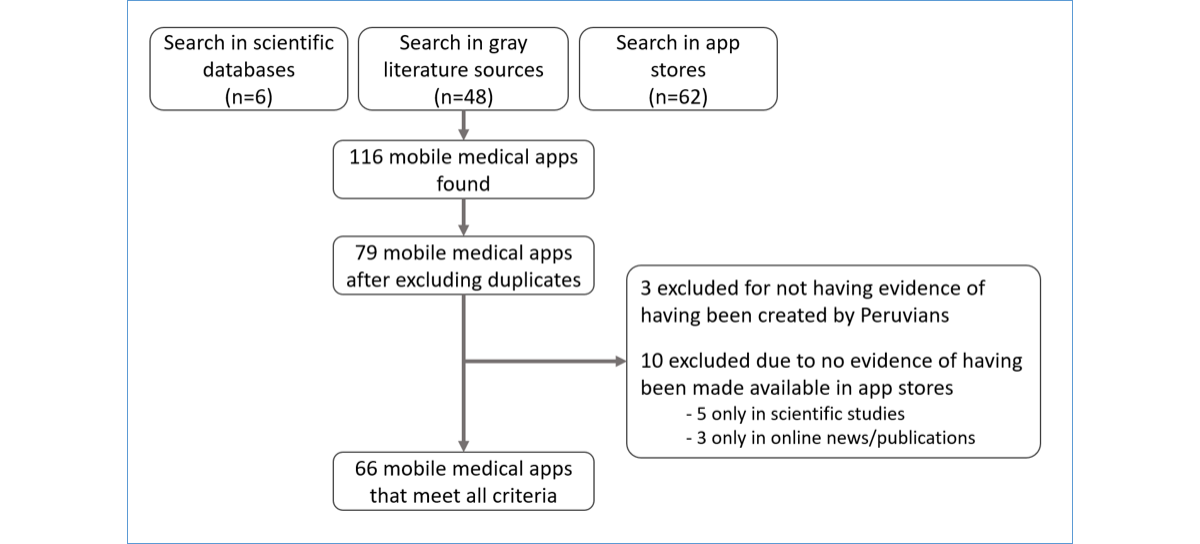
Flowchart of Peruvian mobile medical app research.

The eligibility criteria were as follows: only apps published in Spanish and with evidence that they were created in Peru were studied. For this purpose, we reviewed information about each app available on the information section of app stores and verified if these were related to Peruvian Institutions (eg, INS, MINSA) or to Peruvian companies or clinics (eg, MAPFRE Perú, Inkafarma, Clinica San Pablo) or if their developers were Peruvian. Apps that were not published openly or that were only used for internal studies and those that could not be accessed to assess their characteristics were discarded (Figure 1).

#### Screening Process

The screening process for identifying apps meeting our inclusion criteria on mobile app stores (Google Play and Apple Store), scientific databases (for scientific literature) and Google Search (for gray literature) was performed by 2 independent authors (LRM and DHEH). To describe the main characteristics of these apps, the authors documented each app’s name, entity that created it, year of last update, description of its functionalities, type of use, target audience, and if there was any evidence of efficacy. A third author compiled a database based on the information provided by the 2 authors, eliminating duplicated information and evaluating if the identified apps and related literature meet our eligibility criteria.

### Review of the Most Used mHealth Apps in Peru

Considering that the Android operating system is used by approximately 89.5% of the population in Peru, we searched for the 10 most downloaded mHealth apps in Peru on the Google Play Store. Apps were chosen based on app market statistics from analytical platforms such as App Annie [14] and SimilarWeb [15], which were reviewed in May 2019. For each app, security and privacy aspects were evaluated through the tool proposed by Dehling et al [16], which has shown good reliability. This tool allows one to evaluate the kind of information collected by mHealth apps, defining whether they are medical or nonmedical apps, and whether they leak, modify, or provide user information to third parties. Thus, we evaluated whether these apps represent a high, low, or no risk to the users, irrespective of whether they were intended to be used by the general public or the health care personnel who might collect patient data. In this study, this tool was applied by 2 independent researchers (JS-V and SE-A) and if there was a doubt it was solved by either consensus or the participation of a third researcher. Additional details about the structure and results obtained using this tool are described in Multimedia Appendix 2.

We also evaluated if these apps had a disclaimer, and whether they declared the terms of use and privacy policies. The quality of the information for each app was assessed by evaluating if it had references, mentioned where the source of information came from, or presented any evidence that the information was accurate. To evaluate all these aspects, we first installed the app, verified all kinds of information required by the app, and then reviewed their terms and conditions of use if these were available.

### Review of the Legal and Regulatory Aspects Related to mHealth in Peru

Peruvian laws and regulations in the area of digital health were reviewed to understand which current legal standards apply or could apply to medical apps. The search terms used in this review were the same as those used in the previous one. The search was performed on *Portal de Estrategia Digital*, which is a web platform designed by the Peruvian Ministry of Health that displays all the contents related to the health information and communications technology norms of Peru [17]. Specifically, we looked for all norms that regulate aspects related to the quality of information provided by health systems, information security, and protection of personal data, as we considered these to be the main aspects that could represent an adequate scenario for the use of mHealth technologies in a useful and safe manner in our country.

### Data Analysis

All the statistical analyses were performed through the Statistical Package Stata version 14.0 for Windows (StataCorp). We performed a descriptive analysis of the characteristics of mHealth apps created in Peru, and evaluated the security and privacy aspects of the 10 most commonly used mHealth apps in Peru. All the quantitative results were presented as categorical data through frequencies and proportions.

## Results

### Mobile Medical Apps Created in Peru

**Figure 2:**
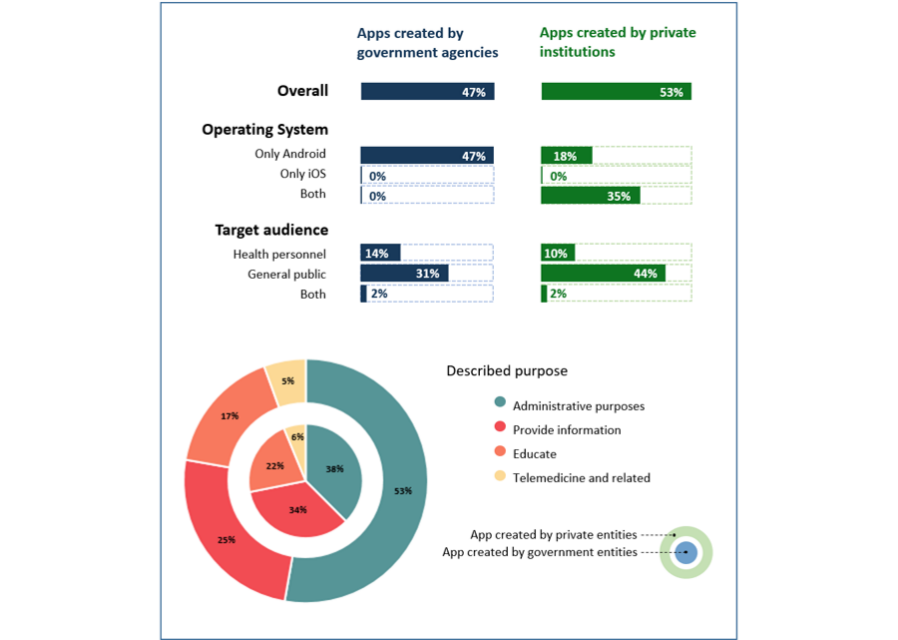
Characteristics of the mobile medical apps created in Peru in the last 5 years (n=66).

We analyzed mHealth apps created in Peru and updated within the last 5 years. A total of 66 apps were found, of which almost half (31/66, 47%) were created by government agencies. The general characteristics are presented in Figure 2. Only 35% (23/66) of these apps could be installed and run on both Android and iOS. In particular, none of the apps created by government agencies (such as Ministry of Health, National Institute of Health; National Health Superintendence) could be run on Apple iOS. Most apps were aimed at the general public for administrative purposes or to provide information on health-related issues.

Concerning the purpose, we identified 4 categories of apps: (1) those created for administrative purposes related, for example, to schedule and manage appointments, pay for health services, field work management, digital health cards; (2) those created to provide information, for example, about the location of health facilities, insurance status, pharmaceutical products, prices, or food composition; (3) those created with the aim of educating, for example, to promote psychological education, or share virtualized books, care guides, and health advices; and finally, (4) those related to telehealth functions to facilitate the offering of virtual and distant communication services among health personnel. The full list of apps is summarized in Multimedia Appendix 3.

We did not find any publications, reports, or articles that provide information on how well the apps work, based on their purpose (effectiveness), how they are perceived by the users (usability), the source of the information they provided, updating frequency, or other mechanisms to ensure the quality of information these apps provide.

Besides the 66 apps we found, it is important to mention that in the scientific literature, there were also other mobile medical apps that were created in Peru, but these were used only for research purposes. Ruiz et al [18] discussed 4 mHealth interventions in Peru, but their apps were not published openly.

### Most Used mHealth Apps in Peru

Table 2 presents data on the 10 most downloaded medical apps in the Peruvian territory. All of them were from private institutions or independent creators. The most downloaded apps were for telehealth services for either symptoms assessment or communication with health professionals. As much as 6 of the 10 apps were aimed at the general public to answer health questions, inform about illnesses, and to monitor conditions such as menstruation and pregnancy. Among the apps aimed at health professionals were those used to consult information about pharmaceutical products, disease codes, and medical concepts.

Concerning the privacy and security aspects of the 10 most downloaded mHealth apps in Peru, which were evaluated using the tool proposed by Dehling et al [16], we found that 5 of 10 (50%) collected medical information such as symptoms, history, doctor, allergies, menstrual cycle, or pregnancy; and 4 of 10 (40%) collected information about sexual history, pregnancy status, or the presence of current illnesses; however, these 4 apps leaked user data and thus could cause embarrassment or affect the employment prospects of their users. In addition, 40% (4/10) of these apps collect information whose loss could cause potential harm to their users (mainly apps that provide telehealth services or estimate conditions/diseases). Finally, 4 of the apps ask for information about the date of birth and contact information (eg, ID in social network, email, or phone number) that are invaluable to third parties, for example, to sell products. Half of these apps did not mention whether security protocols are followed to protect the information they collect (Table 3).

Regarding the quality of information provided by these apps, only 4 mentioned the source of the information provided. The others neither had any bibliographical references nor mentioned if a health professional participates within the operating team.

**Table 2:** Top 10 most downloaded medical apps in the Peruvian territory as of May 2019.

Rank	App	App rating (stars)	Description	Target audience	Purpose
1	Ada – Tu guía de salud	4.7	Ada, your health guide. Ada can help you get accurate health reports. Ada will evaluate your symptoms and, in an instant, will give you relevant information and the next steps to take.	General Public	Telehealth service
2	Chat Médico mediQuo	4.5	Make your consultations to specialized doctors in an easy and simple way and through your smartphone. Chat with our doctors and specialists who will answer questions instantly.	General public	Telehealth service
3	Vademecum Perú	3.8	The app allows access to information on medicines, laboratories, pathologies, interactions, pharmacological index, and therapeutic index. It gathers all the relevant information related to pharmaceutical products.	Health personnel	Provide information
4	CIE10 Español	4.3	This app contains the entire International Classification of Diseases (ICD-10) in one place.	Health personnel	Provide information
5	Calendario Menstrual	4.7	An app that helps and assists women in monitoring their periods, cycles, ovulation, and the possibility of pregnancy (fertile days).	General public	Management purposes
6	Mi Calendario de Embarazo	4.8	Get detailed information about your baby’s development in each week of pregnancy. It includes daily information about pregnancy, feeding advice, control of weight gain per month and belly growth, weight gain calculator, and much more.	General public	Information/management purposes
7	Anatomy Learning - 3D Atlas	4.6	Real-time 3D Atlas. Dissect and learn anatomy.	General public	Education
8	Disorder & Diseases Dictionary	4.7	Medical dictionary that provides more than 1000 definitions of diseases.	General public	Provide information
9	Medical Terminology Dictionary	4.4	This dictionary of medical terms includes more than 10,000 entries with anatomical, pathological, diagnostic definitions, etc.	Health personnel	Provide information
10	Diagnósticos de Enfermería	4.4 stars	Nanda Nursing Diagnostics 2018-2020	Health personnel	Provide information

**Table 3:** Aspects of security, privacy, and quality of the information in the 10 most downloaded medical apps in Peru (data as of May 2019).

Aspects			Apps, n (%)
**Security and privacy^a^**			
	**Specificity: Health specificity of information available to apps**		
		Standard	4 (40)
		Nonstandard	1 (10)
		Medical	5 (50)
	**Leaks: Potential damage through information leaks**		
		None	6 (60)
		Low	0 (0)
		High	4 (40)
	**Change: Potential damage through manipulation (change) of information**		
		None	1 (10)
		Low	1 (10)
		High	8 (80)
	**Loss: Potential damage through loss of information**		
		None	4 (40)
		Low	2 (20)
		High	4 (40)
	**Value: Value of information to third parties**		
		None	4 (40)
		Low	2 (20)
		High	4 (40)
**Quality of information**			
	Include a disclaimer to report limitations		5 (50)
	Mention the source of the provided information		4 (40)
	Mention how often the information is updated		0 (0)

^a^Analyzed using the security and privacy assessment tool for mHealth apps proposed by Dehling et al [16].

### Legal and Regulatory Aspects Related to mHealth in Peru

Among the Peruvian norms related to electronic health (eHealth) and health information technology services that could be applied to Peruvian mHealth apps, we found the following laws.

#### Law No. 29733: Law on the Protection of Personal Data

It establishes that “one of the rights that all people have is the right to have their personal data protected, so that such information would not be used in a way that harms them, threatening the dignity and integrity of the people” [19]. Therefore, the necessary guarantees must be given to control the use of personal data, regardless of how they are used. mHealth apps developed in Peru have the obligation to comply with such provisions.

#### Law No. 29459: Law of Pharmaceutical Products, Medical Devices, and Health Products

The law defines a medical device as any instrument, device, implement, machine, reagent, in vitro calibrator, computer application, material or another similar article provided to be used either alone or in combination with human beings for functions of [20]:

- Diagnosis, prevention, monitoring, treatment, or relief of a disease.
- Diagnosis, monitoring, treatment, relief, or compensation of an injury.
- Research, replacement, or modification of the anatomy or a physiological process.
- Support or maintenance of life.
- Control of conception.
- Disinfection of medical devices.

Although this law does not specifically mention mobile app, it does mention that any computer application that declares any of these functions as intended use will be considered a medical device. It is requested that in order to approve a medical device, it must have an approval from a foreign entity (such as the FDA) or follow a national approval process.

#### Administrative Directive No. 230: Administrative Directive That Establishes the Standards and Technical Criteria for the Development of Health Information Systems

This administrative directive is only for establishments and dependencies of the Ministry of Health [21]. It aims to establish standards for the development of information systems and mentions that health sector information systems could be of 2 types:

1. Administrative information systems that integrate data from administrative processes and procedures such as planning, budgeting, logistics, finance, human resources, and
2. Health information systems that integrate data on the processes and procedures involved in people’s health; health insurance; epidemics and health emergencies; environmental health and food safety; health intelligence; pharmaceutical and medical products, medical devices, and pharmaceutical establishments; human resources in health; infrastructure and sanitary equipment; and health research and technologies.

#### Law No. 30421: Telehealth Framework Law

Telehealth has been regulated in Peru since 2016 and its definition was recently modified according to Legislative Decree 1303 [22]. It is defined as the provision of distance health services by trained health personnel. The Law defines 4 areas of Telehealth app: Telemedicine, Telemanagement, Teletraining, and TeleECI (education, communication, and information to the general population). Telehealth regulations so far do not specifically mention the use of mobile apps as a tool or component necessary to achieve the objectives.

## Discussion

### Summary of Evidence

According to our results, there are many issues on mHealth apps created in Peru with regard to security and privacy of data, quality of information provided, and evidence of their effectiveness that need to be evaluated and regulated. Because the current Peruvian norms do not fully cover all these aspects, there is a need to create and implement a specific Peruvian regulatory framework to mHealth apps.

### Characteristics of Peruvian mHealth Apps and Need for Their Regulation

#### Security and Privacy Aspects

Concerning the security and privacy aspects, we found that 40% (4/10) of the most used mHealth apps in Peru included data whose manipulation, leak, or loss could induce potential harm or damage to their users, for example, affecting their reputation and employment prospects or misorient aspects related to their personal health care. Despite this issue, we found that many of these apps do not clearly mention how the data they collect will be used in their terms and conditions policies. In addition, the information related to the security and privacy aspects of these apps is usually presented in long texts with complex structures, so most users would likely accept these terms and conditions without reviewing them [23,24]. In this context, there is a need to ensure that all apps include a plan or strategy to avoid or prevent the change, leak, or loss of information (either provided or asked) as well as implement other security and privacy features of personal data management according to the Peruvian norms.

#### Quality of Information

Concerning the quality of information presented, we found that among the most used mHealth apps in Peru, only about half report the source of information they provide; notably, none of them reported whether this information is regularly updated. This could represent a potential risk to users of these apps, as most apps are oriented toward general public that do not have enough background to evaluate the quality of information they receive. Indeed, one study showed that about 30%-40% of Peruvian adult population do not have adequate knowledge and competence to access, understand, and apply information received about health topics [25]. This situation suggests the need to ensure that all of these apps include the references used to elaborate the information they provide, or at least indicate that this information is provided by health professionals or subject matter experts.

#### Evidence of Usability and Effectiveness

We found that none of the Peruvian mHealth apps available on app stores have been evaluated in published scientific studies about their usability and effectiveness. Usually the quality of these apps is evaluated in the app stores by a 5-star rating system and reviews from their users that give an indication about their usability, which is then used as a feedback to their developers to improve the characteristics of these apps. However, this information does not necessarily reflect the actual effectiveness of these apps in relation to their mentioned purpose of use. In this context, there is a need to consolidate a list of mHealth apps that provide information not only about the user’s satisfaction level (star rating) but also about the studies that support their effectiveness in relation to their mentioned purpose.

In addition, we found that none of Peruvian mHealth apps that have been evaluated in scientific studies or developed in technology innovation events was currently available on app stores. This is likely because these apps were developed for research purposes and their developers may not have the intention or capabilities to continue their use for commercial purposes which could sustain their development and use in the future. In this context, it is important for business incubators, founders, or interested companies to identify these apps and support their developers until these apps could be made sustainable.

### Proposal of a Legal Framework About Peruvian mHealth Apps

#### The Experience of the FDA

In the United States, the US FDA published in February 2015 a guide that details how it regulates mHealth apps [3]. According to this, the FDA only regulates medical apps that meet the following definition of a medical device: (1) Those that are intended to be used as accessories for a medical device (eg, extension of a vital signs monitor or an app that controls an infusion pump); and (2) Those that transform a mobile platform into a medical device by including accessories (eg, app that connects a glucose strip so that the mobile phone functions as a glucometer) and by providing a specific diagnosis or treatment (eg, apps that provide a diagnosis when monitoring clinical parameters).

It is important to mention that this control is determined by the intended use that the app declares. For this, it uses a risk-based approach (from level I [low risk] to level III [high risk]). Among the examples of major risks (level III), it is worth mentioning apps such as arrhythmia detector or electrocardiogram or blood pressure measurement system. Examples of low-risk apps are those linked to electronic medical records, patient self-care, and patient communication with their health care provider.

#### The Experience of the European Union

In Europe, the EU, like the FDA, regulates the use of mHealth apps if these are considered medical devices. According to the EU regulation, an mHealth app can be a medical device if it contemplates the intention of being used by people with at least one of the following purposes: (1) diagnosis, prevention, control, treatment, or relief of a disease; (2) the diagnosis, control, treatment, relief, or compensation of an injury or deficiency; (3) examination, replacement, or alteration of the anatomical structure or a physiological process; and (4) contraception. In this situation, a same app with different purposes may or may not considered a medical device according to its use; however, its design, use, and data collection policies should adhere to the proper medical device regulations of EU.

EU regulations propose that the use of medical devices in Europe do not need state approval; however, they must comply with the so-called Essential Safety and Performance Requirements of the European Medical Device Directive, which varies according to the potential risk of the product [26]. Therefore, they need technical documentation that contains a report on risk analysis and a clinical evaluation. In addition, the EU states are in charge of monitoring the product continuously after commercialization. According to these regulations, medical devices are classified into 4 categories based on risk level: class I risk (low-risk potential), class IIa (medium risk potential), class IIb (higher risk), and class III (particularly high-risk potential) [27].

#### Proposed Regulatory Framework

Considering all aforementioned aspects, a regulatory framework for mHealth apps in Peru could be based on the current Peruvian regulations with certain considerations according to international experiences.

As proposed by international regulatory agencies, the main step to regulate mHealth apps would be to determine whether they meet the definition of a medical device [28,29]. For this, we could use the Law of Medical Devices (Law No. 29459) which points out that all medical devices in Peru must obtain the approval of a foreign entity (eg, the FDA) or follow a national approval process (which since its implementation in 2017, despite multiple requests, has only managed to register a few). This is an important issue because it must be decided whether every mobile app needs a comprehensive approval, or as international agencies do, whether each kind of risk requires its own form of approval.

In addition, as the environment of the mHealth apps experiences constant innovation and re-design, the legal framework should include a not-so-exhaustive list of the characteristics of Peruvian mHealth apps considered as medical devices and also those that are not in order to provide clarity and assistance in decision making. This list should include characteristics such as security and privacy aspects, the quality of information provided, and the existence of available evidence (preferably, published in scientific journals) about the usability and effectiveness of these apps [30].

Apps that do not meet the definition of a medical device must also be regulated. The FDA stresses the importance of long-term monitoring of apps that are not considered medical devices, such as those designed to help patients to control their disease, those that help organize health information, and those that help to access information. In Peru, mHealth apps that are not considered medical devices could be considered as an information system and thus could be regulated by Administrative Directive 230, which establishes the technical standards and criteria for health information system–based apps created by public institutions.

Unlike Law No. 29459, whose scope applies to the entire national territory, Directive 230 only applies to establishments of the Ministry of Health, so it would be necessary to create a regulation whose scope applies to the entire national level, irrespective of the health subsector the app belongs to.

### Conclusions and Recommendations

In Peru, the rapid increase of the use of mobile technology in health has led to a widespread creation of mHealth apps. However, their use presents a risk to users, as there are lack of strategies or norms to manage security and privacy aspects, quality of information provided, and available evidence about their usability and effectiveness. Given that in Peru the existing norms do not exhaustively cover all these aspects, we recommend the formulation and promulgation of a legal framework specific to mHealth apps based on international models and current national legislation. It should include the creation of a national repository of mHealth apps that provides information related to the aspects discussed in this work. In the same way, we suggest performing studies that evaluate the use and impact of these apps on patients and health professionals, to establish their associated benefits and risks.

## Appendix

Multimedia Appendix 1 Search algorithms.

Multimedia Appendix 2 Security and privacy tool to mhealth apps.

Multimedia Appendix 3 Database of Mobile Apps created in Perú in the last 5 years.

## Abbreviations

EU: European Union
FDA: Food and Drug Administration
