# Supplementary material for: Need for the Development of a Specific Regulatory Framework for Evaluation of Mobile Health Apps in Peru: Systematic Search on App Stores and Content Analysis
Source: JMIR Mhealth Uhealth. 2020 Jul 10;8(7):e16753. doi: 10.2196/16753 (PMC7382017; doi:10.2196/16753)
Supplement: Multimedia Appendix 1 [file mhealth_v8i7e16753_app1.docx]

**SEARCH ALGORITHMS**

**For scientific literature**

***For Scopus***

TITLE-ABS-KEY (Peru OR Peruvian) AND TITLE-ABS-KEY (mobile OR mhealth OR app OR application OR mobile software OR cellular phone OR mobile phone OR Android OR IOS AND TITLE-ABS-KEY (patient care OR self-care OR health services OR diagnosis OR treatment OR education OR information OR insurance OR attention OR consultation)

***For Pubmed and other scientific databases***

(Peru OR Peruvian) AND (mobile OR mhealth OR app OR application OR mobile software OR cellular phone OR mobile phone OR Android OR IOS) AND (patient care OR self-care OR health services OR diagnosis OR treatment OR education OR information OR insurance OR attention OR consultation)

**For grey literature**

For search on grey literature on Google Search we used multiple combinations of words that included:

1. At least one word related to Peru or Peruvian institutions (Eg. Peru, Peruvian, MINSA, DIGESA, INS, DIRESA, DIRIS, SIS, SuSalud, EsSalud, Ministry of Health, National Institute of Health) and
2. At least one word related mobile devices (mobile, mhealth, app, application, mobile software, cellular phone, mobile phone, Android, IOs), and
3. At least one word related with health or care (patient care, self-care, health services, diagnosis, treatment, education, information, insurance, attention, consultation).

Examples:

“Peru apps information health”

“MINSA application diagnosis”

“INS app education”
